# Intron Variant Cause *DICER1* Syndrome With Pleuropulmonary Blastoma

**DOI:** 10.1155/humu/8884636

**Published:** 2025-04-01

**Authors:** Rujin Tian, Yixiao Li, Lin Zhong, Haozheng Zhang, Zhongtao Gai, Dong Wang, Li Song, Kaihui Zhang

**Affiliations:** ^1^Pediatric Research Institute, Children's Hospital Affiliated to Shandong University, Jinan, China; ^2^Department of Ophthalmology, Shandong Provincial Hospital, Cheeloo College of Medicine, Shandong University, Jinan, China; ^3^Hematology and Oncology Department, Children's Hospital Affiliated to Shandong University, Jinan, China

**Keywords:** *DICER1* gene, *DICER1* syndrome, minigene splicing assay, pleuropulmonary blastoma

## Abstract

*DICER1* syndrome (OMIM 601200) is a rare autosomal dominant familial tumor susceptibility disorder with heterozygous *DICER1* germline mutations. The most common tumor in clinical practice is pleuropulmonary blastoma. Pleuropulmonary blastoma is a rare pediatric lung tumor that begins during fetal lung development and is part of an inherited tumor syndrome. We found a patient with pleuropulmonary blastoma in clinical practice and performed whole-exome testing on him and his parents. The mutation is located at *DICER1* gene, c. 1510-16G>A. The tested person has a heterozygous variation at this locus. The tested person's father has no variation at this locus, while the tested person's mother has a heterozygous variation at this locus. According to the ACMG guidelines, this mutation has been preliminarily determined as clinically significant (uncertain) PM2_Supporting: The frequency of this supporting variation in the normal population database is unknown; there is no report of correlation for this locus in the literature database, and the ClinVar database does not feature this locus. In point pathogenicity analysis results, analysis of splicing was carried out by Sanger sequencing and RT-PCR from peripheral blood and a minigene splicing assay, both of which showed a deletion of exon 10 resulting from the c. 1510-16G>A variant at the mRNA level. Bioinformatic analysis of the reported c. 1510-16G>A variant suggests that the variant is pathogenic. Based on the clinical characteristics of the patient and the functional verification of the gene variants, our pediatricians have finally diagnosed the infant with pleuropulmonary blastoma (OMIM 601200). Our findings expand the mutation spectrum leading to *DICER1* deficiency-related diseases and provide accurate information for genetic counseling.

## 1. Introduction

**Figure 1:**
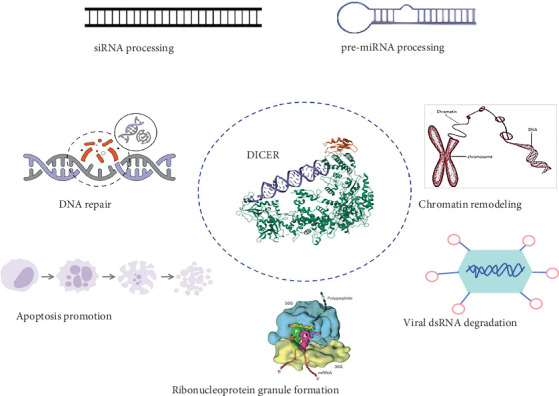
Functions of *DICER1*. Illustrative scheme showing the canonical (siRNA and pre-miRNA processing) and noncanonical functions (chromatin remodeling, DNA repair, ribonucleoprotein granule formation, apoptosis promotion, and viral dsRNA degradation) of DICER.

*DICER1* syndrome (OMIM 601200) is a rare autosomal dominant tumor susceptibility syndrome caused by heterozygous germline pathogenic alterations in the *DICER1* gene on chromosome 14q32.1 [1]. This syndrome increases the lifetime risk of developing a variety of unique benign and malignant tumors, including pleuropulmonary blastoma, thyroid tumors, kidney tumors, renal anaplastic sarcoma, head and neck tumors, central nervous system tumors, soft tissue sarcomas, and female genital tract tumors [2]. Mutations in the *DICER1* gene can increase the pathogenicity of certain tumors. In childhood, they can lead to the development of pleuropulmonary blastoma (PPB) [3]. PPB (OMIM 601200) represents the most prevalent primary lung malignancy encountered in pediatric patients [4]. PPB is mainly diagnosed in infancy and early childhood, with most cases occurring before the age of seven [5]. *DICER1* protein is a member of the Ribonuclease (RNase) III family, which is involved in the production of small functional RNA molecules such as microRNAs (miRNAs) and small interfering RNAs (siRNAs). These small RNAs act as negative regulators of gene expression [6, 7]. *DICER1* has an RNA helicase motif that contains a DEXH box at its amino terminus and an RNA-binding motif at its carboxyl terminus. *DICER1*, also known as helicase multiplicity of infection (MOI), is necessary for RNA interference and short-time RNA (stRNA) pathways to produce active small RNA components that inhibit gene expression. It cleaves naturally occurring long double-stranded RNAs and short hairpin precursor miRNAs to produce short interfering RNAs (siRNAs) and mature miRNAs. siRNAs and miRNAs serve as guide molecules, directing the RNA-induced silencing complex (RISC) to bind to complementary RNAs, either degrading them or preventing their translation [8, 9]. *DICER1* loss in developing lung epithelium disrupts the miRNA-mediated control of diffusible growth factors (Figure 1), leading to the stimulation of mesenchymal cell proliferation [10].

## 2. Patient

### 2.1. Medical History

A male child, aged 3 years and 11 months, was admitted to our hospital's Respiratory Intervention Department on January 31, 2023, due to “chest pain for 1 day.” The child had no significant past medical history and no family history of tumors.

#### 2.1.1. Physical Examination

Examination results are as follows: temperature: 36.1°C, respiratory rate: 24 breaths/min, heart rate: 120 beats/min, and weight: 19 kg. The child appeared well-developed and moderately nourished but exhibited tachypnea. On auscultation, breath sounds were diminished in the left lung and coarse in the remaining lung fields, with no dry or wet rales. Heart rhythm was regular, and heart sounds were strong. The abdomen was soft and not distended, with no palpable liver or spleen below the costal margin.

#### 2.1.2. Diagnostic Workup

##### 2.1.2.1. Chest Ultrasound

A solid, medium-echo mass measuring approximately 13.5 × 11.9 × 8.4 cm was detected in the left thoracic cavity. The mass appeared multinodular with relatively clear boundaries and heterogeneous internal echoes, suggesting a space-occupying lesion in the left thoracic cavity.

##### 2.1.2.2. Chest CT (Plain + Contrast-Enhanced)

A large mass was observed in the left thoracic cavity, with maximum dimensions of approximately 99.7 × 84.2 × 117.5 mm. The internal density was uneven, with small patchy areas of slightly lower density. During the arterial phase of contrast-enhanced scanning, the mass showed significant heterogeneous enhancement, with multiple tortuous enhancing vessels visible inside. The origin of these vessels was unclear. The degree of enhancement was higher during the venous phase than during the arterial phase. Local invasion and thickening of the left chest wall were noted, with unclear boundaries between the intercostal muscle layer and the lesion. These findings indicated a space-occupying lesion in the left thoracic cavity.

##### 2.1.2.3. Orbital Contrast-Enhanced MRI

An abnormal signal nodule was observed in the left orbital region, with suspected involvement of the adjacent frontal dura mater, raising the possibility of metastatic disease. Pathological findings: On February 2, 2023, an ultrasound-guided biopsy of the left thoracic mass was performed. Pathological examination revealed a mesenchymal-derived malignant tumor, characterized by oval to short spindle-shaped cells with significant atypia. Based on histological morphology and immunohistochemical results, the diagnosis was consistent with PPB. Immunohistochemistry results: Vimentin (+), CD99 (-), Desmin (+), Myogenin (+), MyoD1 (+), CD45 (-), STAT6 (-), P53 (partially +), CK (+), BCOR (+), CCNB3 (-), INI-1 (+), Ki-67 (+70-95%), and Syn (-).

### 2.2. Treatment and Management

Based on the patient's clinical manifestations, imaging data, and pathological results from puncture biopsy, we employed a treatment regimen combining adjuvant chemotherapy, surgery, and postoperative adjuvant chemotherapy. The chemotherapy regimen consists of ifosfamide (IFO), vincristine (VCR), dactinomycin (AMD), and doxorubicin (DOXO), administered over cycles lasting 3 weeks each. For the first two cycles, the dosing is as follows:

- IFO is administered at a dose of 3 g/m^2^ once daily for 2 consecutive days.
- VCR is given at a dose of 1.5 mg/m^2^ once daily for 3 consecutive days.
- AMD is administered at a dose of 1.5 mg/m^2^ once daily, but only on 1 day of the cycle.
- DOXO is administered at a dose of 30 mg/m^2^ once daily for 2 consecutive days.

Starting from the third cycle, the dosing of VCR is reduced to 1.5 mg/m^2^ once daily, administered on only 1 day of the cycle, while the dosing of the other drugs (IFO, AMD, and DOXO) remains unchanged.

From the fifth cycle onward, further adjustments are made:

- IFO is administered at a dose of 3 g/m^2^ once daily, but only on 1 day of the cycle.
- VCR is given at a dose of 1.5 mg/m^2^ once daily, administered on only 1 day of the cycle.
- AMD is administered at a dose of 1.5 mg/m^2^ once per cycle.
- DOXO is discontinued from this point onward.

After four cycles of chemotherapy, a chest CT scan of the child showed improvement in the left-sided tumor lesion, indicating that surgical intervention was feasible. Under general anesthesia, a left upper lobectomy and partial resection of the left chest wall were performed. The surgery was successful, and postoperative pathological examination revealed PPB (Type III) with post-chemotherapy changes. Most of the tumor tissue showed necrosis, fibrous tissue hyperplasia, and foamy cell reaction, with some areas exhibiting differentiation into rhabdomyosarcoma or chondrosarcoma. Additionally, the bronchial resection margin and the left chest wall specimens showed no evidence of tumor tissue.

The child continued to receive eight additional cycles of chemotherapy. By the 30th week, imaging studies confirmed no residual tumor, indicating that the child had achieved complete remission (CR) and was in a tumor-free state.

## 3. Material and Methods

### 3.1. Compliance With Ethical Standards

This work was approved by the Medical Ethics Committee of the Children's Hospital affiliated to Shandong University (ethics approval no. QLET-IRB/P-2021037-1.). Clinical and laboratory examinations were performed on the proband after the written informed consent was obtained from her parents or guardians. All procedures in this study were performed in accordance with the Helsinki Declaration.

### 3.2. Next-Generation Sequencing (NGS) and Variant Calling

DNA was extracted from blood samples of the proband and her parents using the QIAamp DNA Blood Midi Kit (Qiagen, Shanghai, China) according to the manufacturer's protocol. The concentration of the extracted DNA was determined using a NanoDrop 2000 UV spectrophotometer (Thermo Fisher, United States). Whole-exome sequencing (WES) was performed using the NovaSeq 6000 platform (Illumina, United States) with Human Exome Probes P039-Exome (MyGenostics, Beijing, China) to screen for variants in the proband. The average exome coverage of the target regions was greater than 95% (>10x coverage; average depth over 100x). Burrows-Wheeler Aligner software (BWA version 0.7.10) was used to align the sequencing data with the human reference genome (hg19). Subsequently, SAM files were converted to BAM. GATK's RealignerTargetCreator and IndelRealigner were applied for local realignment, GATK's BaseRecalibrator was used for quality score recalibration, and variants were jointly called using GATK's HaplotypeCaller in “GENOTYPE_GIVEN_ALLELES” mode. SNPs and indels were then filtered using GATK's VariantFiltration, and ANNOVAR was used to annotate the variants. To remove common variants (suballelic frequency > 5%), variant frequencies were checked against 1000 Genomes, ExAC, gnomAD, ESP6500, and in-house databases. Potentially disease-causing variants associated with the patient's standardized HPO phenotype were prioritized in this study. The pathogenicity of novel variants was evaluated using SIFT, PolyPhen-2, MutationTaster, SpliceAI, and REVEL. Furthermore, variations reported in HGMD (http://www.hgmd.cf.ac.uk) and ClinVar (http://www.ncbi.nlm.nih.gov/clinvar) will be further analyzed. The variants identified in this study were classified according to the 2015 American College of Medical Genetics and Genomics (ACMG) guidelines [11].

### 3.3. Sanger Sequencing for Variant Verification

The potentially pathogenic variants identified in the proband through WES using specific primers were further validated by Sanger sequencing. The reference sequence utilized was *DICER1* chr14:95583048. Primer sets for Sanger validation were designed using Primer Premier v5.0 software. PCR amplification was conducted with AmpliTaq Gold 360 DNA polymerase from Applied Biosystems. After purifying the PCR products via agarose gel electrophoresis and gel extraction, sequencing was performed on an ABI Prism 3700 automated sequencer (Applied Biosystems, Foster City, CA, United States).

### 3.4. RNA Extraction, cDNA Acquisition, and Sanger Sequencing

According to the instruction manual, the total RNA extracted from the peripheral blood of the proband is stored in the TRIzol reagent (Invitrogen, United States). Firstly, peripheral blood cells are lysed by mixing them with a TRIzol reagent, a monophasic solution containing guanidine isothiocyanate, phenol, *β*-mercaptoethanol, and other components. Guanidine isothiocyanate, as a chaotropic agent, is a potent protein denaturant that can dissolve proteins, eliminate their secondary structure, degrade their superstructure, and rapidly separate nucleoproteins from nucleic acids. Due to the efficient inhibition of RNase activity, disruption of cells, and dissolution of cellular components during sample homogenization, the TRIzol reagent maintains the integrity of RNA. Subsequently, chloroform is added to accelerate the separation of the organic and aqueous phases. The upper layer is the aqueous phase with a pH of around 5.1, where only RNA molecules remain, while DNA molecules precipitate at the interface between the phenol and the solution. The RNA is retained in the aqueous phase and recovered through precipitation with isopropanol. The concentration of the obtained ribonucleic acid is measured using a NanoDrop 2000 UV spectrophotometer (Thermo Fisher Scientific, United States). Following the instruction manual, cDNA is acquired from RNA reverse transcription of the proband's peripheral blood using the Takara PrimeScript RT Kit (Takara). Sequencing fragments, including variations in the *DICER1* gene, were amplified from the cDNA of the proband using the forward primer *DICER1*-F (5⁣′- GTTTGAAAGCGTTGAGTGGTATAA -3⁣′) and the reverse primer *DICER1*-R (5⁣′- CGTGTTGATTGTGACTCGTGG -3⁣′). Later, possible cDNA alterations caused by the c.3453C>T variant were detected through Sanger sequencing, as described above.

### 3.5. Minigene Splicing Assay

To verify the splicing effects potentially caused by the c. 1510-16G>A (p.?) variant, a minigene splicing assay was conducted in vitro. The minigene regions of the *DICER1* gene, spanning exon 8, intron, exon 9, intron, and exon 10, were amplified from the gDNA of the control group using a forward primer (5⁣′-ttggtaccgagctcggatccATTGATAAAGGAAGCTGGCAAACA-3⁣′) with a BamHI restriction site and a reverse primer (5⁣′-acgggccctctagactcgagCTATTGATGTGTCCAATGGCCG-3⁣′) with an XhoI restriction site. The target gene was amplified using the ClonExpress II One-Step Cloning Kit (Vazyme, Nanjing, China) and ligated into the pcDNA3.1 vector. The cloned wild-type plasmid was validated using Sanger sequencing. Mutant plasmids were created by recombining mutant fragments obtained with mutagenesis primers *DICER1*MF (5⁣′-GTGCTTTTCTTGGTaGATTTTTTTTCAGGTACTTAGGAAAT-3⁣′) and *DICER1*MR (5⁣′-TCtACCAAGAAAAGCACTTCTAAGAAAATTTC-3⁣′). The mutation sequence of the plasmid was verified by Sanger sequencing, and the mutated recombinant plasmid was transferred into HEK293T cells using Lipofectamine 3000 (Invitrogen) according to the manufacturer's instructions. Total RNA was extracted from cells cultured for 48 h using the TRIzol reagent (Invitrogen, United States). RT-PCR was performed using the MiniRT-F primer pair (5⁣′ GAGTCCAGGAGCCAGTCTGATA 3⁣′) and MiniRT-R (5⁣′ GGCAAATCAAAACGAACCACC 3⁣′). The amplified PCR fragments were analyzed by agarose gel electrophoresis, and the isoforms were identified by Sanger sequencing.

## 4. Results

### 4.1. Genetic Analysis

**Figure 2:**
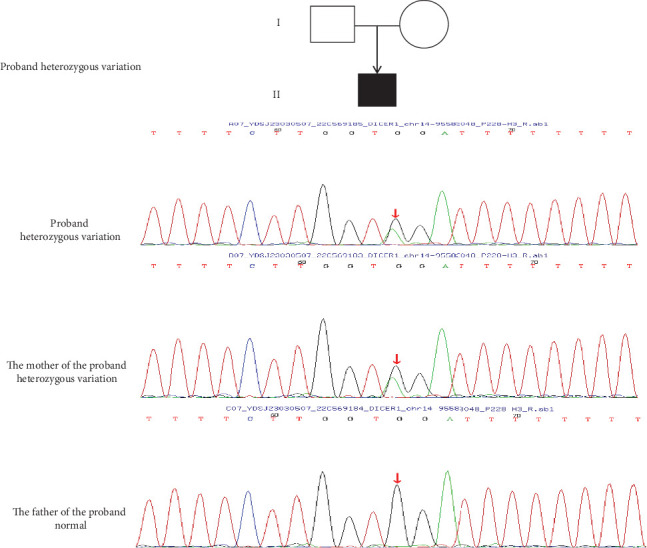
The Sanger sequencing results of the variant c.1510-16G>A. The Sanger sequencing showed that c.1510-16G>A was maternally inherited.

Based on the patient's clinical presentation, imaging examination, pathological biopsy, and immunohistochemical diagnosis, the patient has been diagnosed with pleuropulmonary blastoma, a rare and highly aggressive malignancy of the lung in children that often involves the pleura and lung, with a familial predisposition. WES conducted on the patient and their parents revealed that the patient carries a heterozygous mutation in the *DCIER1* gene (c.1510-16G>A) (Figure 2). This discovery is of significant importance for understanding the patient's disease pathogenesis and developing individualized treatment plans.

### 4.2. Identification of Alternative Splicing by Extracting RNA From Patient's Peripheral Blood

**Figure 3:**
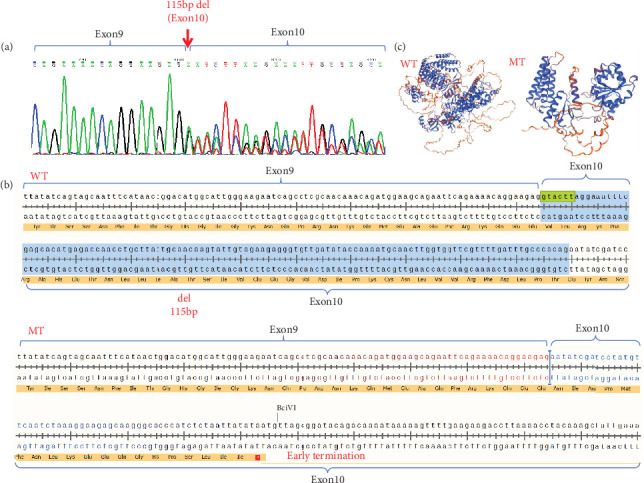
Splicing study of *DICER1* c.1510-16G>A. (a) The Sanger sequencing of RT-PCR products based on peripheral blood samples of the proband. (b) Schematic of splicing sequence for DICER1 c.1510-16G>A; blue nucleotides were the deletion of exon 10 (115 bp). The amino acids corresponding to the base sequence encoding are highlighted in yellow. Base deletion can lead to nonsense mutations WT: wild type; MT: mutation type. (c) Use the SWISS-MODEL website (https://swissmodel.expasy.org/) to predict the tertiary structure of proteins corresponding to amino acid sequences.

We extracted RNA from the patient's peripheral blood and obtained cDNA through RT-PCR. Using cDNA as a template, we designed validation primers that encompassed exons flanking the intron mutation. The PCR products were then verified through Sanger sequencing. The sequencing results indicated the coexistence of both the wild-type normal splice variant and the mutant abnormal splice variant. The *DICER1* (c.1510-16G>A) mutation led to abnormal splicing occurring in exon 10, resulting in a partial deletion of exon 10 (Figure 3a).

### 4.3. Splicing Study of *DICER1* (c.1510-16G>A) by Minigene Assay

**Figure 4:**
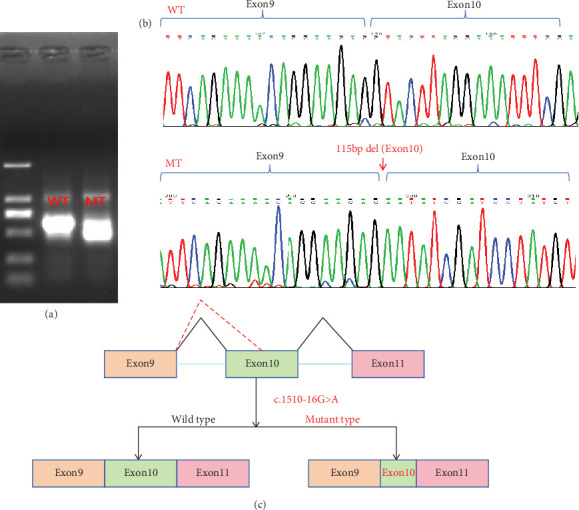
Splicing study of *DICER1* c.1510-16G>A by minigene assay. (a) Agarose gel electrophoresis results of RT-PCR for the plasmid expression. (b) The Sanger sequencing of RT-PCR for the plasmid expression. (c) Schematic of splicing for c.1510-16G>A. WT: wild type; MT: mutation type.

We constructed both wild-type and point mutant plasmids of the *DICER1* gene and transfected these two plasmids into 293T cells. RNA was then extracted from these cells. The cellular RNA underwent reverse transcription while being validated with specific primers. We performed Sanger sequencing on the PCR products, and the results showed that the wild-type plasmid produced a normal splice variant, while the mutant plasmid resulted in abnormal splicing with a deletion of 115 bp in exon 10, which is consistent with the previous findings from the patient's blood study (Figure 4).

### 4.4. Analyzing the Variant Pathogenicity According to ACMG

According to the ACMG guidelines, the variant c.1510-16G>A (p.?) is deemed a pathogenic variation (pathogenic), specifically categorized under PVS1_Strong: The mutation triggers altered splicing, ultimately leading to premature termination of translation. Through familial validation analysis, it was discovered that the subject's father did not have the variation at the corresponding locus, whereas the subject's mother exhibited a heterozygous variation at that locus. However, the mother does not manifest the disease, which may be associated with incomplete penetrance or expressivity of the *DICER1* gene.

## 5. Discussion

We conducted WES for the proband and his/her parents, and the sequencing results revealed an intron mutation in *DICER1* (c.1510-16G>A). We validated this finding using Sanger sequencing, which confirmed the presence of this mutation in the intron of the proband. To investigate whether this mutation in the intron led to the generation of abnormal splice variants, we performed sequencing analysis on the peripheral blood of the patient. The sequencing results indicated the presence of abnormal splicing of the mRNA encoding the *DICER1* protein. To further verify that this alternative splicing was caused by the *DICER1* (c.1510-16G>A) mutation rather than other factors, we employed the minigene approach for detection [12]. The results showed that abnormal splicing occurred in 293T cells transfected with the mutant plasmid, and the splicing pattern was consistent with that observed in the patient's peripheral blood. Therefore, we concluded that this mutation leads to the formation of abnormal splice variants.

**Figure 5:**
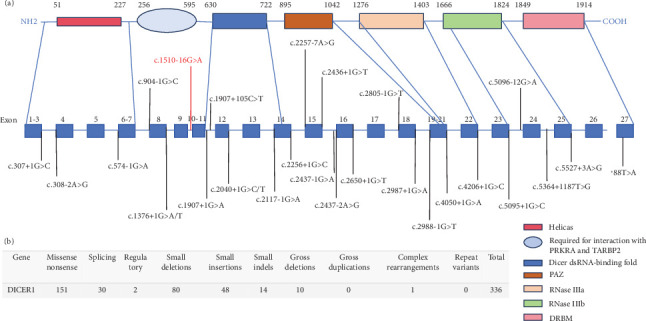
Schematic diagram for *DICER1* and landscape of intronic mutation. *DICER1* contains 7 important domains helicase, required for interaction with PRKRA and TARBP2, dicer dsRNA-binding fold, PAZ, RNaseIIIa, RNaseIIIb, and DRBM. The reported 27 intronic mutation in DICER1 is shown over the diagram. The variants reported are shown in black, and the variant identified in this study is in red.

*DICER1* germline mutations are typically inherited in an autosomal dominant manner [13]. As previously reported, mutations in the *DICER1* gene are shown in the figure below (Figure 5), with data sourced from the Human Gene Mutation Database (https://www.hgmd.cf.ac.uk/ac/index.php). The variant (c.1510-16G>A) which could result in the deletion of exon 10 (115 bp) in the *DICER1* cDNA predicted to result in a truncated protein was located in the helicase C-terminal (Figure 3c). Germline mutations (i.e., inherited mutations from parents) in the *DICER1* gene are associated with several rare hereditary tumor syndromes, such as *DICER1* syndrome (also known as pleuropulmonary blastoma family tumor predisposition syndrome (PPB-FTPS)). These syndromes increase the risk of developing multiple types of tumors in patients, including pleuropulmonary blastoma, ovarian sex cord-stromal tumors, renal cancer, and others [13–15]. Due to the crucial role of *DICER1* in the RNAi pathway, its mutations may lead to abnormal biosynthesis of small RNAs, thereby affecting the regulation of gene expression. This abnormal regulation may promote abnormal cell proliferation and tumorigenesis [16].

The proband experienced a base mutation in the intron, resulting in the appearance of alternative splicing bodies, and a 115 bp deletion in the tenth exon, leading to a nonsense mutation (Figure 3b). In affected families, germline loss-of-function mutations in the RNase IIIb domain of *DICER1* can lead to the development of PPB, and premature termination mutations in introns can affect the RNase IIIb domain of the *DICER1* protein [10].

The tumor susceptibility in patients with *DICER1*-related diseases is inherited in an autosomal dominant manner. However, the penetrance of *DICER1* mutations is quite low (approximately 15%) [6], which explains why many carriers never develop tumors. This may account for the observation that both the mother and the child carry a pathogenic intron mutation, yet only the child develops the disease while the mother does not. In our hospital, we identified four children with *DICER1* syndrome. Among them, three inherited the *DICER1* mutation from their phenotypically normal mothers, while the inheritance pattern of the fourth case could not be verified due to unavailability of the maternal sample (unpublished data).

The molecular diagnosis for PPB patients is pivotal not only for the proband but also for the family, as to provide precise genetic counseling and execute the prenatal diagnosis to deliver a healthy child.

### 5.1. The Limitation and Attention

PPB typically presents with late-onset symptoms and rarely includes hemoptysis, which can lead to misdiagnosis. Symptoms include persistent cough, dyspnea, fever, lung infections such as pneumonia, wheezing, chest or abdominal pain, anorexia, unexplained weight loss, and fatigue require vigilance. Mutations in the *DICER1* gene are a significant risk factor for the development of PPB, and patients with a family history of *DICER1* mutations or PPB should be particularly alert. When performing prenatal genetic diagnosis, carrier parents may potentially affect the birth of a healthy child who does not suffer from the disease.

## Data Availability

The data that support the findings of this study are available on request from the corresponding author. The data are not publicly available due to privacy or ethical restrictions.
